# Green Biologics: Harnessing the Power of Plants to Produce Pharmaceuticals

**DOI:** 10.3390/ijms242417575

**Published:** 2023-12-17

**Authors:** Gergana Zahmanova, Alaa A. A. Aljabali, Katerina Takova, George Minkov, Murtaza M. Tambuwala, Ivan Minkov, George P. Lomonossoff

**Affiliations:** 1 Department of Plant Physiology and Molecular Biology, University of Plovdiv, 4000 Plovdiv, Bulgaria; katerina.takova@uni-plovdiv.bg (K.T.);; 2 Center of Plant Systems Biology and Biotechnology, 4000 Plovdiv, Bulgaria; 3 Department of Pharmaceutics and Pharmaceutical Technology, Faculty of Pharmacy, Yarmouk University, Irbid 21163, Jordan; alaaj@yu.edu.jo; 4 Lincoln Medical School, University of Lincoln, Brayford Pool Campus, Lincoln LN6 7TS, UK; mtambuwala@lincoln.ac.uk; 5 Institute of Molecular Biology and Biotechnologies, 4108 Markovo, Bulgaria; 6 Department of Biochemistry and Metabolism, John Innes Centre, Norwich NR4 7UH, UK; george.lomonossoff@jic.ac.uk

**Keywords:** plant-derived biologics, plant-based expression systems, plant molecular farming, synthetic biology, genome editing, vaccines, antibodies, therapeutic enzymes, virus-like particles

## Abstract

Plants are increasingly used for the production of high-quality biological molecules for use as pharmaceuticals and biomaterials in industry. Plants have proved that they can produce life-saving therapeutic proteins (Elelyso™—Gaucher’s disease treatment, ZMapp™—anti-Ebola monoclonal antibodies, seasonal flu vaccine, Covifenz™—SARS-CoV-2 virus-like particle vaccine); however, some of these therapeutic proteins are difficult to bring to market, which leads to serious difficulties for the manufacturing companies. The closure of one of the leading companies in the sector (the Canadian biotech company Medicago Inc., producer of Covifenz) as a result of the withdrawal of investments from the parent company has led to the serious question: What is hindering the exploitation of plant-made biologics to improve health outcomes? Exploring the vast potential of plants as biological factories, this review provides an updated perspective on plant-derived biologics (PDB). A key focus is placed on the advancements in plant-based expression systems and highlighting cutting-edge technologies that streamline the production of complex protein-based biologics. The versatility of plant-derived biologics across diverse fields, such as human and animal health, industry, and agriculture, is emphasized. This review also meticulously examines regulatory considerations specific to plant-derived biologics, shedding light on the disparities faced compared to biologics produced in other systems.

## 1. Introduction

Protein-based biologics are the fastest-growing class of pharmaceutical products, manufactured from engineered biological sources. Plants can be engineered to produce various types of biologics (antibodies, vaccines, enzymes, therapeutic proteins, hormones, and cytokines), as well as recombinant proteins for cosmetics, food, and the chemical industry, in addition to research and diagnostic purposes. The process is known as plant molecular farming (PMF) [1,2,3,4]. PMF offers a safe, cost-effective, and scalable production of unique multimeric proteins, ensuring fast and global-scale deployment of biologics and other valuable recombinant proteins [5]. These advantages make plants an efficient alternative to the traditional expression systems such as bacterial, yeast, insect, and mammalian cells, which cannot fully satisfy global needs for biologics and industrial proteins [6,7,8]. The successful implementation of well-established practices, such as plant cell engineering and optimization of biosynthetic pathways through cell medium improvement or genome editing, increased yields by using different methods of expression, or improving downstream processing (DSP), has led to optimization and maturation of PMF processes [9,10,11]. Companies producing plant-derived recombinant proteins for industrial purposes have seen sustainable success overall. In the pharmaceutical sector, however, this success has been limited to just a few notable examples (Elelyso™, ELFABRIO^TM^, and growth factors). This is most likely due to a lack of regulatory frameworks, an inability of plant-based expression systems to compete with established industrial platforms, and the reluctance of large pharmaceutical companies to reorganize their manufacturing processes [12,13]. Despite the larger demand for biologics and the proven effectiveness of plant-derived biologics, big pharma companies have not adapted their production to plant-based expression.

This review seeks to identify some of the factors that influence this and the challenges associated with the production and utilization of plant-derived biologics, including low yields, expensive DSP, and potential safety concerns. In this context, “plant-derived biologics” primarily signifies biopharmaceuticals and proteins resulting from the genetic modification of plants. This process involves engineering plants to express therapeutic proteins, antibodies, vaccines, and biosynthetic enzymes that are not naturally present. Genetic modification allows precise control over gene expression, optimization of metabolic pathways, and customization of protein properties to fulfill therapeutic, industrial, or agricultural needs. Innovative strategies, such as genome editing, plant glycosylation pathway remodeling, and synthetic biology, are discussed in the context of enhancing production efficiency and product efficacy. By addressing recent advancements, regulatory considerations, challenges, and prospects, this review seeks to establish itself as an indispensable reference for those interested in harnessing the immense possibilities offered by plant-derived biologics across various domains.

## 2. Types of Plant-Derived Biologics

Plant-derived protein-based biologics represent a diverse array of molecules that can be produced using plants as an expression system [14,15,16]. They often have highly complex structures containing additional moieties (glycocarbohydrates and fatty acids) and can be divided into several major categories: monoclonal antibodies (mAbs), vaccines, enzymes for replacement therapies, receptor modulators, and bioactive small molecules [17]. Table 1 presents the main commercial achievements in the production of plant-derived biologics. The diverse range of recombinant proteins presented the potential of plants as versatile platforms for producing recombinant proteins.

### 2.1. Antibodies and Antibody Fragments

Plant-based expression systems have been leveraged for producing monoclonal antibodies and antibody fragments, known as “plantibodies.” Their applications include therapeutics, diagnostics, and research. Plantibodies have already successfully targeted infectious agents, cancer biomarkers, and therapeutic entities (Table 1) [33]. During the 2014 Ebola outbreak, for instance, the plant-based production of ZMapp (a cocktail of monoclonal antibodies) significantly increased the survival rate of infected patients [34], though the small number of patients treated makes a statistical evaluation of efficacy difficult. Plantibodies offer potential advantages in terms of reduced production costs, scalability, and customization.

### 2.2. Vaccines and VLPs

Plants present an appealing platform for vaccine production due to their ability to express immunogenic viral and bacterial antigens, as well as highly organized virus-like particles (VLPs) [35,36,37,38,39]. VLPs are self-assembling virus coat proteins resembling viruses but devoid of genetic material, which makes them safe for use in vaccines and nanoparticle production. Plant-derived VLPs and chimeric VLPs (composed of structural proteins or immunogenic epitopes from different viruses) are used as immune modulators and self-adjuvants in order to provoke strong immune responses against different viral diseases, as well as others such as cancer, allergies, and autoimmune diseases [5,40,41,42,43]. Plant-derived vaccines offer a multitude of benefits, including reduced production costs, improved stability, minimal cold chain requirements, and the possibility of oral delivery. Notably, edible vaccines have emerged, utilizing genetically engineered plants to express vaccine antigens. This innovative approach holds great promise for transforming vaccine delivery, particularly in developing countries where oral administration can eliminate the need for injections and cold chain storage. However, a major disadvantage is controlling the vaccination dose [8,36,44].

Furthermore, VLPs can be used as nanoparticles for drug delivery systems [45].

### 2.3. Therapeutic Enzymes

Plants efficiently produce various recombinant enzymes with clinically improved profiles. For example, Elelyso^TM^ (β-glucocerebrosidase) has an improved profile compared to its CHO-made counterpart because the final plant-derived β-glucocerebrosidase contains terminal mannose residues, which are a key factor in the success of enzyme replacement therapy for Gaucher’s disease treatment [46,47]. Preclinical and clinical studies have shown promise for plant-based production of insulin and pegunigalsidase alpha (Table 1). Further, plant-made enzymes can be delivered orally, due to the cellulose wall of plant cells that makes them resistant to degradation [48]. Recombinant protein medications are shielded from stomach acids and digestive enzymes by the plant cell wall polymers, which have β 1,4 and β 1,6-glycosidic linkages that are resistant to hydrolysis [49]. Commensal microorganisms in the intestinal epithelium break down the plant cell wall, releasing the bioencapsulated recombinant proteins, which are recognized by the gut-associated lymphoid tissues (GALT) and induce an adaptive immune response [50,51]. Utilization of plant expression systems offers numerous advantages and has demonstrated promising outcomes in both preclinical and clinical studies, presenting a potential alternative to traditional biologic production methods [52,53,54]. Notably, plant-based expression systems have facilitated the cost-effective production of complex recombinant proteins, revolutionizing human and animal health management [5,55,56].

### 2.4. Receptor Modulators

Plant-based expression systems have been successfully used for the production of various small polypeptides and glycoproteins involved in the regulation processes in mammalian cells, such as cytokines and hormones [57,58]. Human growth hormone (hGH) produced in *N. benthamiana* plants demonstrated its biological activity in a hypophysectomized rat [58]. Cytokines are signaling proteins that help control inflammation in the body and can be used in the treatment of cancer, immune disorders, and various other related diseases. Erythropoietin [59], IL-2 [60], IL-4 [61], IL-12 [62], IL-13 [63], IL-18 [64], cardiotrophin 1 [65], human granulocyte-macrophage colony-stimulating factor (GM-CSF) [66,67,68,69], tumor necrosis factor-alpha (TNF) [70], interferon-alpha [71], human fibroblast growth factor 8b [72], and insulin-like growth factor 1 [73] have been expressed in different plant species using various methods for increasing recombinant protein yield and stability.

### 2.5. Small Molecules

Plants are a natural source of many medicinal compounds based on secondary metabolites, such as triterpenoids, alkaloids, and phenolics. These are synthesized through multi-step biosynthetic pathways involving a variety of different enzyme activities, and the active products have a highly specific stereochemistry. Such medicinal compounds can be extracted from the native species; however, in many cases, propagation of the plants and the low yields of extractable compounds and their subsequent purification make this an expensive procedure. The alternative of complete chemical synthesis is generally impractical due to the complex structures of the compounds. For these reasons, attention has turned to the detailed characterization of the biosynthetic pathways for plant-derived metabolites with the aim of reconstructing them in more tractable organisms through the co-expression of the relevant biosynthetic enzymes. Such an approach also allows variants of naturally produced molecules to be produced through the combinatorial expression of different enzymes.

A dramatic early example of this approach was the reconstruction of the biosynthetic pathway for the anti-malarial compound artemisinin in yeast [74]. Subsequently, the relevant enzymes have been transferred to other organisms, including plants more tractable than *Artemisia annua*, especially *N. benthamiana* (reviewed by Zhao et al., 2022) [75]. Though stable transformation has been used in plants, the “go-to” method is transient expression of the relevant enzymes. Not only is this much quicker than stable transformation, but it also allows a combinatorial approach for making a wide variety of related compounds that have differing bioactivities [76,77]. This ability has been exploited to great effect in the case of triterpenes [78,79], culminating in the transient combinatorial expression of 16 enzymes in *N. benthamiana* to produce a saponin molecule suitable for further bioengineering to produce adjuvants for use with vaccines [80]. This approach has also been used in studies to elucidate the biosynthetic pathway of the anticancer drugs vinblastine [81,82] and paclitaxel [83]. Though, at the time of writing, none of the molecules produced in this way have made it to deployment in medicine, the technology has great promise.

### 2.6. Bioactive Proteins from Plants

Plant-derived biologics also encompass bioactive molecules, such as lectins. Lectins are natural proteins that can bind carbohydrates that are highly specific for the sugar groups of other molecules. Some of the lectins have potent antimicrobial activity through binding to carbohydrates on microbial surfaces and inducing changes in cell permeability and pore formation [84]. Their biological activities make them useful as microbicides, antitumor agents, and vaccine adjuvants [85,86,87]. Mistletoe lectins (ML-I, ML-II, and ML-III) were transiently expressed in *N. benthamiana* and demonstrated anticancer activity [88].

## 3. Strengths, Weaknesses, Opportunities, and Threats (SWOT) Analysis of Biologics

One of the primary advantages of harnessing plant-derived biologics lies in their inherent safety profile and reduced unwanted immunogenicity, which paves the way for enhanced patient tolerance and minimized adverse effects [89]. The plant glycan moieties’ immunogenicity has been the subject of extensive studies, and various animal models have been investigated to elucidate the immunogenicity of plant-derived glycoproteins [90,91]. The (1,2) xylose and (1,3) fucose structures have been recognized as cross-reactive carbohydrate determinants as a result of the discovery of IgE antibodies in allergy patients that cross-react with these structures on glycoproteins from a variety of species [92]. Plant-derived taliglucerase alfa (TGA), Protalix, contains tri-mannose glycoform with the addition of β(1,2) xylose and α(1,3) fucose, which are present at above 90% of the total glycan pool. No overtly adverse effects that could be related to these N-glycan residues have been observed in a clinical trial with TG involving healthy human volunteers, and no anti-drug antibodies have been found [93].

Additionally, whole plants and plant cell suspension cultures, which are free from animal pathogens and toxins, are suitable for oral delivery of biologics without purification or with minimal purification [48,94]. Production of oral biopharmaceuticals in edible plant tissues has proven to be efficacious in several clinical vaccinations for disease prevention [95,96,97,98]. The ability of plant cell walls to protect the plant-made biologic from enzymatic degradation in the gastrointestinal tract and the ability of these biologics to reach the lymphoid tissue in the gut in their active form can lead to the induction of oral tolerance, the prevention of unwanted immune responses, and the prevention of allergic responses [99,100,101,102]. Furthermore, the cost-effective production and scalability of these biopharmaceuticals present an opportunity to address the escalating healthcare costs and global disparities in access to essential medications [103,104]. Plant-based biologics’ extraordinary versatility allows for their precise customization and tailoring in order to address diverse therapeutic needs as shown in Table 2. By capitalizing on the inherent adaptability of plants, scientists can usher in a new era of personalized medicine, where targeted therapies are tailored to individual patients, boosting treatment outcomes and improving overall patient well-being [105]. In light of these remarkable advancements, it becomes increasingly evident that plant-derived biologics represent a transformative force in the field of biopharmaceuticals, offering a multifaceted solution to the pressing challenges faced by modern medicine [106]. Noteworthy stories, such as the development of plant-based antibodies or VLPs for treating cancer and the utilization of plant-produced vaccines for infectious diseases, underscore the efficacy and potential of these biologics [107,108,109,110,111,112,113,114]. Moreover, the environmental sustainability of plant-derived biologics deserves recognition. Their production systems boast lower carbon footprints, reduced energy requirements, and decreased reliance on non-renewable resources compared to other biological production methods. Addressing regulatory and safety considerations is paramount. Adherence to guidelines established by regulatory agencies ensures the approval and quality control of these biologics; however, unwavering vigilance is still necessary to guarantee safety and efficacy throughout the production process. Further, they also have weaknesses in terms of time consumption, variable yields, regulatory considerations, and protein degradation. Threats include intellectual property barriers, competition, and public perception. Continued research, development, and regulatory efforts are crucial to overcome these challenges and fully realize the potential of plant-derived biologics [15,115].

## 4. Manufacturing of Plant-Based Production Systems

**Figure 1:**
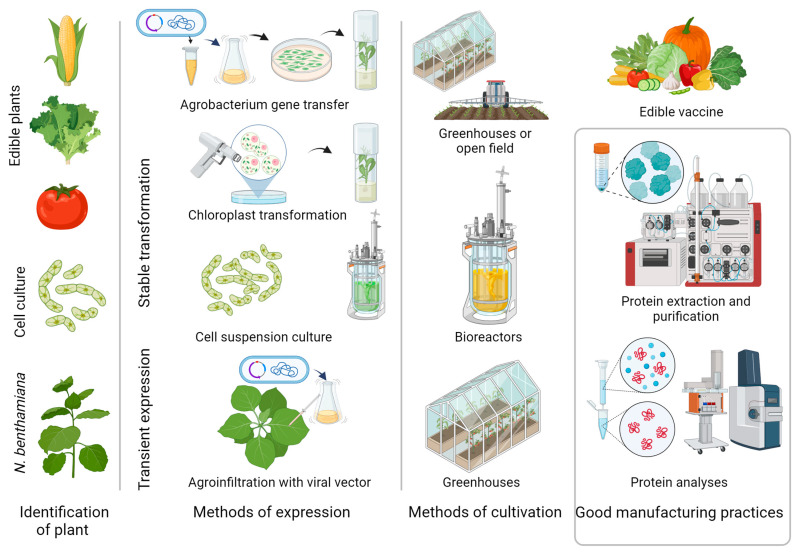
Main steps in the production of biologics in plants. Valuable recombinant proteins are produced in stable transgenic whole plants (nuclear or chloroplast) using stable transgenic plant cell cultures and transient expression via agroinfiltration or modified plant viruses.

Many of the original technical weaknesses of PMF, such as low yields, low recovery of recombinant proteins after downstream processing, and the impact of specific glycosylation in plants, have been overcome in some cases. Introducing technologies, such as stable plastid transformation, plant cell cultures, transient expression, agrobacterium infiltration, modeling of the plant glycosylation pathway, plant genome editing for improving the biosynthetic pathways in plants, increasing protein stability, and the production in current Good Manufacturing Practice (cGMP), will lead to scaling up the manufacturing of functional, unique recombinant proteins in plants [7,116,117,118,119,120,121]. The main steps in the development of PDB are presented in Figure 1.

The extraordinary variety of plant species with specific useful characteristics enables the development of a wide variety of plant production systems [12]. Tobacco, maize, rice, potato, tomato, carrot, and lettuce are commonly used plant species for stable (nucleus or chloroplast) transformation [120,122,123,124,125,126]. Plant cell culture systems such as ProCellEx^®^ and tobacco cell cultures (BY-2 and NT-1) covered the criteria for a high yield, precise control of environments and cell growth conditions, production according to good manufacturing practices, and regulatory compliance [127]. These advantages of plant cell systems have brought them to market with two products (β-glucocerebrosidase and a vaccine against Newcastle disease virus) approved, respectively, by the US Food and Drug Administration (FDA) and the US Department of Agriculture (USDA) [27,128]. Plant cell suspension cultures are a connecting link between plants and current commercial cell-based production platforms. Furthermore, higher plants are not the only ones used for the production of therapeutic protein cell suspension systems: *Chlamydomonas reinhardtii* (alga), *Lemna minor* (duckweed), and *Physcomitrella patens* (moss) [129]. In addition to expression within living cells, a cell-free system based on BY-2 cells harvested in the exponential growth phase has been investigated as a means of synthesizing proteins. Yields of up to 270 μg/mL have been obtained when producing the yellow fluorescent protein (eYFP) using this approach [130].

The tobacco relative, *Nicotiana benthamiana,* is the most frequently used plant in molecular farming to produce recombinant proteins using transient expression [131,132,133]. *N. benthamiana* is characterized by the accumulation of a large biomass in a short period, reduced gene silencing, and being the most suitable plant species for transient expression using plant virus-based vectors and agrobacterium infiltration [35,134,135,136,137]. Utilization of plant viruses, such as tobacco mosaic virus (TMV) or cowpea mosaic virus (CPMV), as full virus vectors for delivering foreign genes into plants began in the 1980s [138,139,140,141]. Later on, these first-generation (gene substitution or insertion vectors) were replaced by deconstructed vector systems [142,143,144]. The deconstructed vectors contain only the viral genome components essential for effective protein expression, allowing the production of up to 80% of total soluble protein (magnICON) [145]. Furthermore, multiple gene expression leads to the accumulation of multi-subunit proteins such as VLPs, IgA, and IgM [146]. Plants with engineered post-translational modifications (glycosylation mutants and expression of recombinant glycosylation enzymes) have been developed for the proper production of recombinant glycoproteins [147,148,149]. New techniques such as CRISPR/Cas9 genome editing have been used to engineer the endogenous N-glycosylation machinery of the plants to generate *N. benthamiana* with deficient α-1,3-fucosyltransferase and β-1,2-xylosyltransferase activity [150]. The advantages and disadvantages of the engineering toolset applied in PMF for the production of recombinant products in plants are shown in Table 3.

## 5. Challenges in Producing and Using Plant-Derived Biologics

### 5.1. Complex Protein Expression

Achieving high-level expression of complex proteins in plants is a significant challenge. Some proteins require intricate folding and assembly processes, which may be difficult to achieve in plant cells. There are two strategies for the expression of multiple recombinant proteins in plants: co-transformation with individual *Agrobacterium* strains, each maintaining a vector to the gene of interest [142]; and construction of multiple recombinant genes, expressed in tandem from a single expression vector [168,169]. Complex recombinant proteins are characterized by much higher accumulation when targeted to the apoplast or endoplasmic reticulum (ER), most likely due to the facilitated folding and glycosylation within the secretory pathway. Moreover, the proteolytic activities at these locations are less active than in cytosol [170,171]. Furthermore, co-expression of recombinant proteins together with chaperones from the same origins as the recombinant gene has been reported to improve protein folding and accumulation. Co-expression of the human chaperone calreticulin (CRT) resulted in improving the protein yield of gp140 HIV-1 antigen and other viral glycoproteins, thus ameliorating the ER stress response [172]. Co-expression of human CRT and SARS-CoV-2 RBD glycoprotein does not lead to the same effect and does not increase the protein yield or improve the folding of RBP [173]. The modification of plant-based expression systems to accommodate such intricate protein structures presents a significant hurdle [174].

### 5.2. Post-Translational Modifications

Post-translational modifications (PTMs) play a crucial role in the functionality and efficacy of many biologics. However, the PTMs found in plants may not always be identical to those found in mammalian systems. Plants perform complex N-glycosylation of recombinant glycoproteins, characterized by plant-specific modifications such as β(1,2)-xylose and core α(1,3)-fucose, but lack pathways for galactosylation, sialylation, core α(1,6)-fucosylation, and bisecting GlcNAc [116]. The type of glycan present in recombinant glycoproteins determines some of their biological properties (immunogenicity, stability, and bioactivity). It is desirable to modify the endogenous N-glycosylation machinery of plants to allow for the synthesis of complex N-glycans. Unmet goals in N-glycan engineering can be met using CRISPR/Cas9-mediated knockout technology. Developing strategies to enhance the fidelity of PTMs in plants remains a key challenge [174,175].

### 5.3. Post-Translational Gene Silencing (PTGS)

Another method for achieving high levels of recombinant protein expression is through repression of the RNA silencing pathway in the host plant. *Arabidopsis thaliana* mutants (*sgs2* and *sgs3*) with repressed silenced PTGS were used to achieve extremely high-level transgene expression of the *β*-glucuronidase (GUS) gene [172]. Improved production of recombinant proteins was reported in *N. benthamiana* by using RNA interference (RNAi) technology, facilitating the knockdown of the *DCL2* and *DCL4* genes [176]. CRISPR/Cas9 technology was successfully used to generate different knockout genes (*RDR6* gene and *ago2* in *N. benthamiana)*, which led to increased recombinant protein accumulation [177,178].

Further, recombinant protein expression can be boosted by co-expression with gene silencing suppressors such as P19 [179,180].

### 5.4. Proteolytic Degradation of Recombinant Proteins

In plant cells, a large group of endogenous proteases are active, performing normal cellular functions but creating a harmful environment for the recombinant proteins [181]. Several proteases are identified as responsible for the proteolytic degradation of recombinant proteins in plants [182]. Using protease gene knockout/knockdown technologies such as CRISPR/Cas9, TALEN, and RNAi, or co-expression of protease inhibitors and pH regulators, can achieve blocking the protease activity [121].

### 5.5. Downstream Processing

Efficient purification and downstream processing of plant-derived biologics can be challenging due to the presence of plant-specific contaminants, such as phenolic compounds, pigments, and polysaccharides. Further, the structural complexity of biologics is a prerequisite for challenges associated with their stability and structural integrity. Biologics are amenable to diverse forms of degradation, including aggregation, isomerization, hydrolysis, deamidation, and oxidation [183,184]. Developing robust and cost-effective purification methods that maintain the structural integrity and biological activity of the biologics is essential [185,186].

## 6. Novel Strategies for Improving Production and Efficacy

### 6.1. Protein Engineering

Utilizing protein engineering techniques, such as codon optimization, gene fusion, or domain shuffling, can enhance the expression and stability of plant-derived biologics [187,188]. By tailoring the protein sequence to better suit the plant expression system, researchers can improve the yield and quality of the produced biologics.

### 6.2. Synthetic Biology Approaches

Advancements in synthetic biology enable the design and construction of customized plant expression systems. By incorporating synthetic gene circuits, promoters, and enhancers, researchers can precisely control gene expression and fine-tune protein production. Synthetic biology approaches offer the potential for optimizing plant-derived biologics’ production and quality [189,190]. Codon optimization of gene coding sequences for recombinant proteins can lead to a 25-fold increase in yield [191]. Using the right combination of genes, gene promoters, terminators, and signals for polyadenylation and constructing effective transgene expression cassettes leads to an increase in the effectiveness of recombinant protein production. The cauliflower mosaic virus (CaMV) 35S promoter, nopaline synthase (NOS) promoter, and ubiquitin promoters are the most commonly used constitutive promoters, being used with preferences in transient expression. Tissue-specific promoters are commonly used for stable transgene expression, especially in cereals [188]. Gene transcription and mRNA processing are greatly impacted by the terminators. Furthermore, mRNA stability and translation can be strongly influenced by 5′- and 3′-untranslated regions (UTRs) [192,193]. The characterization of various promising 5′-UTRs and 3′-UTRs and their use in the construction of expression cassettes led to an increase in the efficiency of recombinant protein production in plants [194].

### 6.3. Metabolic Engineering

Metabolic engineering aims to modify the metabolic pathways within plant cells to enhance the production of desired biologics. By manipulating enzyme activities, precursor availability, and regulatory mechanisms, researchers can improve yields and redirect metabolic flux toward the desired products. Metabolic engineering holds promise for improving the overall productivity of plant-based biologics [188,195].

### 6.4. Advancing Plant Molecular Biology

Further understanding of plant molecular biology and cellular processes will enable researchers to develop more efficient plant expression systems. Exploring the intricacies of protein folding, post-translational modifications, and regulatory mechanisms within plant cells will facilitate the production of high-quality plant-derived biologics. Integrating omics technologies, such as genomics, transcriptomics, proteomics, and metabolomics, can provide comprehensive insights into plant biologics production. These approaches can aid in identifying key regulatory factors, improving target gene selection, and optimizing metabolic pathways for enhanced production and efficacy [196].

### 6.5. Field Scale-Up and Commercialization

Scaling up production processes and achieving commercial viability are critical for realizing the full potential of plant-derived biologics. Developing robust, large-scale cultivation systems and cost-effective downstream processing methods will be essential for the industrial-scale production of plant-derived biologics [197,198,199]. The remarkable ability of tobacco cell suspension cultures for protein biosynthesis is demonstrated by the optimized BY-2 lysate, which produced yields that were 15-fold higher than any other eukaryotic batch-based cell-free system producing comparable proteins [200]. LenioBio Ltd. is the company that has brought the BYL system to the market [201]. The companies have mostly concentrated on the production of higher-added-value biopharmaceuticals. Three primary categories of protein products have surfaced in this context: growth factors and cytokines, antibodies, and replacement human proteins like human serum albumin, insulin, β-glucocerebrosidase, pegunigalsidase alfa, and gastric lipase (Table 1) [202,203,204]. The most successful products produced by plants are recombinant antibodies, which can be easily purified, accumulated to large levels (>100 mg/kg fresh plant weight), and have straightforward binding assays to confirm their activity. However, the accumulation of antibodies in plants is still significantly below the yields attained in Chinese hamster ovary (CHO) cells. There are some niche areas (the need for the presence of plant-specific glycans, edible vaccines and therapeutics for veterinary application, emergency vaccine production, and animal-free protein production) in which plants provide unique qualities that CHO cells and other expression systems cannot match [205].

Although plant expression systems have found their niche and demonstrated, albeit in rare cases, their economic and technological advantages over established commercial productions, the industry is not inclined to invest in a new type of capacity and switch its production to plant-based production. This is partly due to the difficulties in downstream processing, the variable quality of the products, and the relatively low yields achieved in the plants. Further work is needed to address any shortcomings of plant expression systems and to make progress along their value chain. This work also includes toxicity research, therapy monitoring, conducting clinical trials, and getting approval from regulatory and health insurance bodies. Bringing more plant-based biopharmaceuticals to market will require closer collaboration between governments, regulatory authorities, and pharmaceutical companies.

## 7. Applications of Plant-Derived Recombinant Proteins

**Figure 2:**
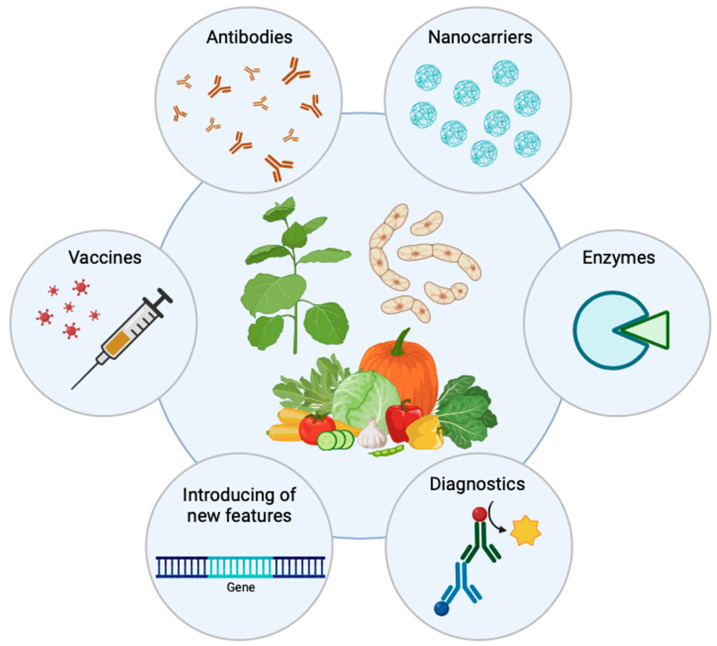
Application of plant-derived biologics.

Plant-derived biologics present a myriad of applications across therapeutic, industrial, and agricultural sectors (Figure 2), intricately linked to the specific attributes of plant-based expression systems [12,206,207,208].

### 7.1. Therapeutic Applications of Plant-Derived Recombinant Proteins for Human and Animal Health

When exploring therapeutic applications, it is imperative to comprehend the distinct demand for plant-based biologics in diverse cultures and nations. Traditional practices in certain regions have long recognized the therapeutic attributes of specific plants, and the incorporation of plant-derived biologics is in harmony with these cultural beliefs. Integrating information on how plant-based therapies have been historically employed in different cultures provides a comprehensive understanding of the necessity for plant-derived biologics. For instance, countries such as China and India have a robust history of utilizing plant extracts in traditional herbal medicine. The transition to plant-derived biologics in these regions serves as an extension of these longstanding practices, establishing a bridge between ancient wisdom and modern biotechnology. While green biologics may not fully replace therapeutics produced via fermentation and mammalian cell cultures, they offer advantages in personalized medicine and the tailored production of monoclonal antibodies (mAbs), therapeutic enzymes, and vaccines for addressing niche diseases. Plant-derived therapeutic proteins find diverse applications in treating conditions like cancer, enzymatic disorders, and genetic disorders [209,210]. Monoclonal antibodies from plant sources play a crucial role in immunotherapy, targeting specific antigens in diseases such as cancer and infectious diseases [211,212]. For instance, plant-based expression systems have been employed to generate mAbs for the treatment of non-Hodgkin lymphoma [213]. Moreover, mAbs hold significant promise in diagnostic applications, serving as diagnostic reagents for specific diseases and research purposes [214,215,216]. The production of recombinant human growth hormone, interferon-α, -β, -γ, interleukins-2, -4, -10, -12, -18, and erythropoietin in plants provides cost-effective and accessible treatment options [54,202]. Plant-based expression systems also facilitate the manufacturing of recombinant viral and bacterial antigens, leading to the development of cost-effective and easily scalable diagnostic reagents and vaccines [217,218,219]. Plant-derived vaccines exhibit potential for preventing a range of infectious diseases, including influenza, severe acute respiratory syndrome-associated coronavirus 2 (SARS-CoV-2), Crimean–Congo hemorrhagic fever virus (CCHFV), Rift Valley fever virus (RVFV), Ebola virus (EBOV), human immunodeficiency virus (HIV-1), dengue virus (DENV), human papillomavirus (HPV), and more [220,221,222,223,224,225,226,227]. In addition, plant-derived biologics find applications in veterinary medicine, producing vaccines against various animal diseases like Newcastle disease, foot-and-mouth disease, avian and swine influenza, rabies, and hepatitis E, offering effective and accessible veterinary solutions [36,228,229,230,231]. Furthermore, the concept of edible vaccines, involving genetic engineering to express vaccine antigens in plants, holds significant promise for oral vaccine delivery, particularly in resource-limited settings [232,233,234]. Plant virus-derived VNPs can be used as nanovesicles for targeted delivery of drugs, nucleic acids, contrast agents, photosensitizers, and enzymes for therapeutic, diagnostic, and agricultural applications [5,235,236,237,238,239].

### 7.2. Biologics Produced in Space

In the context of agricultural and industrial applications, particular regions employ specific agricultural practices that can derive benefits from plant-derived biologics. Illuminating these practices and elucidating how plant-derived biologics align with or enhance traditional methods adds depth to the discussion. Plant molecular farming can be carried out on site with less sophisticated infrastructure than traditional biologics production. This could address problems with therapeutic protein production and distribution aboard space stations or even on long-term missions to the Moon or Mars. Experiments in this field have already been carried out, such as a NASA-funded study using transgenic lettuce and potato leaves to manufacture growth factors and hormones for astronauts [240].

### 7.3. Industrial and Agricultural Applications of Plant-Derived Recombinant Proteins

Plants can be engineered to produce enzymes and other proteins with industrial applications. These plant-derived recombinant proteins may find use in sectors such as textile manufacturing, the paper and pulp industry, and biofuel production. For instance, plant-derived cellulases are employed in the breakdown of plant biomass for bioethanol production, providing sustainable and eco-friendly alternatives to conventional industrial processes [241]. Plant molecular farming offers a sustainable and cost-effective approach to the production of biopolymers, such as biodegradable plastics and biofibers. Plant expression systems allow for the production of biopolymers, such as animal proteins (collagen, keratin, silk, and elastin), which are characterized by great flexibility, toughness, strength, and biocompatibility. CollPlant Inc., Ness Ziona, an Israel-based company, has created a tobacco cell line producing recombinant human collagen, achieving a yield of 200 mg/kg leaf material [242]. Further, plant-derived recombinant human collagen can be used in cosmetics. The first spider silk proteins were produced in plants by Scheller et al., who were able to achieve up to 2% TSP of silk protein in transgenic potato tubers and tobacco leaves [243,244]. These plant-derived biopolymers have potential applications in biomedical engineering, cosmetics, and as adhesives, contributing to the development of environmentally friendly materials [245,246].

Plant-derived biologics can be employed in animal feed formulations to enhance nutritional content, improve feed efficiency, and promote animal health. For instance, plant-derived enzymes can be added to animal feed to improve digestion and nutrient utilization in livestock, supporting efficient and sustainable animal production [247].

## 8. Regulatory Considerations for Plant-Derived Biologics

Regulatory considerations play a crucial role in the development and approval of plant-derived biologics. As these biologics are unique, they require specific regulatory frameworks that take into account their distinct characteristics [248]. Equivalence to materials produced through other systems is a complex issue with far-reaching implications. In the context of regulatory considerations for plant-derived biologics, this issue is multifaceted and demands a thorough analysis. First and foremost, regulatory agencies, such as the US FDA and the European Medicines Agency, play a pivotal role in ensuring the safety and efficacy of plant-derived biologics. The requirement for specific regulatory frameworks is grounded in the unique nature of these biologics, which distinguishes them from other production systems. One key aspect that regulatory agencies scrutinize during evaluation is the source of the plant expression system. This includes a detailed examination of the plant species, genetic modifications, and expression vectors utilized in the production of these biologics. The aim here is to assess the potential risks associated with the use of plant-based expression systems. Given the diversity of plant species and the inherent complexities of genetic modifications, this is not merely a matter of equivalence but rather a nuanced assessment of the uniqueness of each plant-derived biologic [249,250].

Furthermore, the regulatory process for plant-derived biologics delves into the comprehensive characterization of these products. This encompasses their structural attributes: purity, potency, and stability. The use of analytical methods and assays is imperative in the evaluation of these parameters, with the goal of ensuring consistency and reproducibility in the manufacturing process. In essence, the issue of equivalence cannot be oversimplified. Each plant-derived biologic is distinct in terms of its source, genetic makeup, and characteristics. Regulatory agencies take a meticulous approach to ascertaining the safety and effectiveness of these biologics by considering the specific attributes of the plant expression system and ensuring rigorous characterization. Consequently, addressing the comment from a scientific and regulatory perspective necessitates a nuanced understanding of the intricacies involved in the assessment of plant-derived biologics [251]. It is important to note that, due to the diversity of plant species and the intricacies of genetic modifications, equivalence is not the sole focus. Rather, the focus is on understanding the unique attributes of each plant-derived biologic and how it aligns with safety and efficacy requirements.

Additionally, the regulatory process for plant-derived biologics involves a thorough characterization of the biologics themselves, encompassing their structure, purity, potency, and stability. Analytical methods and assays are employed to evaluate these parameters and ensure consistency and reproducibility in the manufacturing process [252].

### 8.1. Current Regulatory Landscape

Before discussing future requirements, it is essential to provide an overview of the current regulatory mechanisms overseeing plant-derived biologics. Regulatory agencies such as the US FDA and the European Medicines Agency play a pivotal role in ensuring the safety and efficacy of these biologics. The existing regulatory framework involves a meticulous examination of various aspects:

**Source of Plant Expression System:** Detailed scrutiny of plant species, genetic modifications, and expression vectors used in production.
**Characterization of Biologics:** Thorough evaluation of structural attributes, purity, potency, and stability.
**Variability in Expression:** Implementation of measures to ensure consistent production and quality despite inherent variability within plants.
**Co-expression of Plant-Specific Proteins/Allergens:** Rigorous characterization is required to identify and mitigate potential risks associated with unintended co-expression.

Plant-derived biologics often face unique regulatory considerations compared to other biologics derived from microbial or mammalian systems. One key difference is the variability in the expression of the desired biologics within plants. Regulatory agencies recognize this variability and require appropriate controls and measures to ensure consistent production and quality of the biologics [253]. Another important aspect is the potential for unintended co-expression of plant-specific proteins or allergens. Regulatory agencies require thorough characterization of the biologics to identify and mitigate any potential risks associated with these co-expressed substances [254]. Furthermore, the regulatory evaluation of plant-derived biologics takes into account the potential environmental impact of the cultivation and production processes. Risk assessments are conducted to ensure that the release of genetically modified plants or their by-products does not pose significant risks to the environment [255].

### 8.2. Future Regulatory Requirements

Having gained a more precise understanding of the current regulatory terrain, we can now navigate the terrain of future requirements. This nuanced approach allows for a meticulous analysis of the evolving regulatory dynamics and the particular needs of plant-derived biologics. It aligns with the industry’s progression towards tailored and case-by-case evaluations as highlighted in Table 4.

In recent years, there have been notable advancements in regulatory approaches for plant-derived biologics. Regulatory agencies have been actively engaged in refining guidelines and incorporating scientific advancements to facilitate the development and approval process. One such advancement is the use of a case-by-case approach in the regulatory evaluation of plant-derived biologics. This approach allows regulators to consider the specific characteristics of each biologic and tailor the requirements accordingly, rather than applying a one-size-fits-all approach [256].

Additionally, regulatory agencies have been working towards harmonization of regulations globally to streamline the development and approval process for plant-derived biologics. Collaboration between regulatory authorities and industry stakeholders has led to the establishment of international guidelines and standards, fostering consistency and efficiency in regulatory evaluations. Furthermore, regulatory considerations for plant-derived biologics encompass a comprehensive evaluation of safety, efficacy, and quality parameters. Distinct regulatory requirements exist to address the unique characteristics of plant-based expression systems. Recent advancements in regulations have focused on tailoring requirements to individual cases and promoting global harmonization. These regulatory frameworks ensure that plant-derived biologics meet the necessary standards for safety, efficacy, and environmental impact, enabling their successful development and approval in the biotechnology industry [257,258].

## 9. Conclusions

In the complex arena of biologics’ expression, the utilization of plants as hosts has emerged as a promising avenue. This amalgamation involves an in-depth exploration of the challenges and strategies related to biologics’ expression in plant systems, drawing on recent scientific findings and advancements. The utilization of plant-based expression systems offers practical advantages. The scalability and cost-effectiveness of plant molecular farming provide unprecedented opportunities for large-scale production. Precision targeting, whether directed towards the apoplast or endoplasmic reticulum, has shown increased protein accumulation, forming a robust foundation for enhanced yields. Additionally, plant-derived biologics exhibit versatility across therapeutic, industrial, and agricultural applications, highlighting their multifaceted potential.

Addressing challenges in achieving high-level expression involves genetic engineering and sophisticated molecular techniques. Approaches such as co-transformation using multiple Agrobacterium strains or constructing multiple recombinant genes in a single expression vector exemplify the meticulous methods scientists employ for optimizing protein expression. The interplay of post-translational modifications, including N-glycosylation engineering through CRISPR/Cas9, opens avenues for tailoring glycan profiles to align with therapeutic requirements. Innovative techniques like synthetic biology and metabolic engineering serve as pillars for precision in controlling gene expression and modifying metabolic pathways.

However, the journey is not without hurdles. Proteolytic degradation within the dynamic environment of plant cells poses a significant challenge, demanding strategies such as gene knockout and protease inhibition for mitigation. Complexities in downstream processing, arising from plant-specific contaminants, necessitate innovative purification methods to ensure the structural integrity of biologics. Regulatory considerations, marked by the uniqueness of plant-derived biologics, demand a nuanced understanding and a case-by-case evaluation for safety and efficacy.

Venturing into the intricate landscape of plant molecular biology, the seamless integration of omics technologies becomes an enlightening companion, revealing the intricacies of protein folding, post-translational modifications, and regulatory mechanisms. The forward march of synthetic biology and metabolic engineering paints a canvas of possibilities, foretelling a future where meticulously crafted plant expression systems are tailored with unprecedented precision.

In conclusion, the realm of biologics’ expression in plants serves as both a testament to progress and a reminder of challenges yet to be overcome. The journey demands scientific acumen and a holistic understanding of the intricate dance between molecular processes, cellular environments, and regulatory landscapes. As we stand on the cusp of transformative advancements, the synthesis of knowledge from diverse perspectives and cutting-edge technologies propels us toward a future where plant-based biologics play an indispensable role in shaping the landscape of medicine, industry, and agriculture.

This comprehensive exploration navigates the intricate landscape encompassing protein-based biologics and the utilization of plants as expression hosts. As the green biologics narrative continues to unfold, the potential of plant-based expression systems is poised to play an indispensable role in shaping the landscape of medicine, industry, and agriculture.

## Figures and Tables

**Table 1:** Advances in plant-based biologics and industrial proteins in the commercial market.

Biologic/Application	Plant/Expression System	Status/Research Findings/Reference/Website Links	Company
Therapeutics			
β-Glucocerebrosidase Gaucher’s disease, enzyme replacement	*Daucus carota*, carrot cell culture, stable gene expression ProCellEx^®^	Elelyso™ has been approved by the USDA and EMA as the first biologic on the market [18]. https://protalix.com/ (accessed 10 October 2023)	Protalix BioTherapeutics Inc., Karmiel, Israel/Pfizer
Pegunigalsidase alfa Fabry disease	ProCellEx^®^ Stable gene expression	ELFABRIO^TM^ has been approved for the USA and EU [19]. https://protalix.com/ (accessed on 10 October 2023)	Protalix BioTherapeutics Inc., Israel
Clinical-grade plant material of the virus-trapping proteins CTB-ACE2 SARS-CoV-2	*Lactuca sativa*, Lettuce stable chloroplast expression	Clinical trial phase I/II of chewing gum containing proteins CTB-ACE2 (angiotensin-converting enzyme 2 fused to the non-toxic cholera toxin subunit B) [20].	University of Pennsylvania
Uricase (PRX 115) Severe Gout	ProCellEx^®^ Stable gene expression	Clinical trial phase I https://protalix.com/( accessed on 10 October 2023)	Protalix BioTherapeutics Inc., Carmiel, Israel
Insulin Diabetes	*Helianthus annuus*, (sunflower)/stable gene expression	Clinical trial phase I/II [21] http://www.sembiosys.com/ (accessed on 9 October 2023)	SemBioSys Genetics Inc., Calgary, Alberta, Canada; in 2012, SemBioSys terminated its operation
Lactoferrin VEN120 Inflammatory bowel disease VEN BETA *E. coli* gastroenteritis	*Oryza sativa*, Transgenic rice seeds, cell culture media	Products on the market https://ventria.com/ (accessed on 9 October 2023)	Ventria Bioscience, Junction City, KS, USA
Allergens bioparticles	*N. benthamiana* Transient expression	Production of high-quality (“natural-like”) allergens and other sophisticated proteins for pharmaceutical purposes https://angany.com/ (accessed on 9 October 2023)	Angani Inc., Québec, QC, Canada
Vaccines			
Influenza VLPs vaccine Seasonal flue	*N. benthamiana* Transient expression	Clinical trial phase III [22]	Medicago Inc., Quebec City, QC, Canada; the company closed in 2023.
Covifenz^®^ SARS-CoV-2 vaccine	*N. benthamiana* Transient expression	Authorized for use by Canada Health after successfully completed clinical trials [23].	Medicago Inc., Quebec City, QC, Canada
KBP-201 with CpG oligonucleotides SARS-CoV-2 vaccine	*N. benthamiana* Transient expression	Clinical trial phase 1/2 https://kbio.com/ (accessed on 10 October 2023)	Kentucky BioProcessing, Owensboro, KY, USA
IBIO-201 IBIO-202 SARS-CoV-2 vaccine	*N. benthamiana* Transient expression	Preclinical studies [24]	iBio Inc., Bryan, TX, USA
Baiya SARS-CoV-2 Vax 1 SARS-CoV-2 vaccine	*N. benthamiana* *Transient expression*	Clinical trial phase 1 ongoing [25] https://baiyaphytopharm.com/ (accessed on 10 October 2023)	Baiya Phytopharm Co., Ltd., Bangkok, Thailand
SARS-CoV-2 RBD vaccine	*Chlamydomonas reinhardtii, algae*	SARS-CoV-2 RBD was evaluated as an oral immunogen in mice. The test immunogen was stable in freeze-dried algae biomass and able to induce mucosal responses [26].	-
HERBAVAC™ CSF Green Marker Classical swine fever virus (CSFV)	*N. benthamiana* Transient expression	Registered by the World Organization for Animal Health (WOAH) http://bioapp.co.kr/eng/ (accessed on 10 October 2023)	BioApplications Inc., Pohang, Republic of Korea
Newcastle disease vaccine (in poultry)	*N. tabacum* sell suspension culture, stable gene expression	The first vaccine produced in plants approved by the US Food and Drug Administration for application in poultry [27].	Dow AgroSciences LLC, Benton County, IN, USA
Oral delivery platform of vaccines	*Chlamydomonas reinhardtii,* TransAlge technology	Edible vaccine https://www.transalgae.com/( accessed on 9 October 2023)	TransAlgae, Rehovot, Israel
Others			
Growth factors, cytokines, lectins anti-CD25 antibody	*N. benthamiana* Transient expression	Research reagents on the market https://ibioinc.com// (accessed on 9 October 2023)	IBio Inc., Bryan, TX, USA
Antibodies, viral proteins, VLPs	*N. benthamiana* Transient expression	Research reagents on the market https://capebiologix.com (accessed on 9 October 2023)	Cape Bio Pharma, South Africa, Cape Town, Africa
Antibodies, enzymes, cytokines VLPs, viral proteins	*N. benthamiana* Transient expression	Research reagents on the market https://www.leafexpressionsystems.com/ (accessed on 10 October 2023)	Leaf Expression Systems, Norwich, UK
Diagnostic antibodies, cytokines, growth factors	*N. benthamiana* Transient expression	Research reagents on the market https://www.agrenvec.es/ (accessed on 9 October 2023)	Agrenvec, Madrid, Spain
Plant virus-like particles, Alternanthera Mosaic Virus	*N. benthamiana* Transient expression	Research reagents on the market https://www.diamante.tech/ (accessed on 9 October 2023)	Diamante Società Benefit, Verona, Italy
Growth factors	*Hordeum vulgare*/Barley grains, stable gene expression	Cosmetics [28] https://www.orfgenetics.com/ (accessed on 10 October 2023)	ORF Genetics, Kópavogur, Iceland
Enzymes	*Zea mays*, Corn, stable gene expression	Industry https://www.infiniteenzymes.com/ (accessed on 10 October 2023) https://www.greenlab.com/#in-production (accessed on 10 October 2023)	Infiniteenzyme Inc., Jonesboro, AR, USA Greenlab, Inc., Jutland, Denmark
Therapeutic antibodies			
Anti-Human rAntibody (BLX-301) Non-Hodgkin’s lymphoma	*Lemna minor*, Duckweed, LEX system Stable gene expression	Phase II; The product and duckweed production system has been abandoned.	Biolex Inc., Pittsboro, NC, USA; the company declared bankruptcy in 2012 [29]
ZMapp™ Anti-Ebola monoclonal antibodies	*N. benthamiana* Transient expression	In randomized, controlled trial, although the estimated effect of ZMapp appeared to be beneficial, the result did not meet the prespecified statistical threshold for efficacy [30]. https://mappbio.com (accessed on 10 October 2023)	Mapp Biotherapeutics, Inc., San Diego, CA, USA
P2G12 HIV-neutralizing human monoclonal antibody 2G12	*Nicotiana tabacum* cv. Petit Havana cv. SR1, stable gene expression	Phase I clinical trial [31]	Pharma-Planta consortium, Fraunhofer IME, Schmallenberg, Germany
Anti-Spike antibody (mAb B38, H4) SARS-CoV-2	*N. benthamiana* Transient expression	Both mAb B38 and H4 demonstrated specific binding to receptor binding domain (RBD) of SARS-CoV-2 and exhibited efficient virus neutralization activity in vitro [32].https://baiyaphytopharm.com/ (accessed on 10 October 2023)	Baiya Phytopahrn, Bangkok, Thailand
Plant-made monoclonal antibody against ricin exposure	vivoXPRESS^®^ plant-based manufacturing system	www.antoxacorp.com (accessed on 10 October 2023) www.swiftpharma.eu (accessed on 10 October 2023)	AntoXa Corporation, Toronto, Ontario, Canada and SwiftPharma, Belgium

**Table 2:** Strengths, weaknesses, opportunities, and threats analysis (SWOT) analysis of plant-derived biologics.

**Strengths**	**Weaknesses**
Low cost: Plants can be grown at a relatively low cost compared to other expression systems. **Profit can be made if the production of recombinant protein is high, the downstream processing is efficient, and there is an opportunity to scale up the production for a short period of time.**	Time consuming: The production process of plant-derived biologics can be time-consuming.
Scalability: Plant-based biologic production can be easily scaled up to meet demand.	Variable yields: The yields of plant-derived biologics can be highly variable.
Complex molecules: Plants can produce complex biological molecules with post-translational modifications.	Regulatory considerations: Plant-derived biologics are subject to regulatory scrutiny and require approval from regulatory agencies.
Safety: Plant-derived biologics are considered safe for human consumption and do not pose a risk of contamination.	Protein degradation: Proteases present in plants can degrade proteins during production.
**Opportunities**	**Threats**
Alternative to traditional expression systems: Plant-derived biologics have clinically improved profiles.	Intellectual property: Intellectual property rights can be a barrier to development and commercialization.
Unmet medical needs: Plant-derived biologics can address unmet medical needs, such as low-cost vaccines for developing countries.	Competition: The field of plant-derived biologics is highly competitive.
Diversification: Using plant-derived biologics diversifies biological production sources.	Public perception: The use of genetically modified plants may face skepticism.

**Table 3:** Comparative analysis of different methods applied in the production of plant-derived biologics.

Method	Description	Advantages	Disadvantages
Stable nuclear transformation [151,152]	Stable integration of the gene of interest into the plant genome, enabling long-term and consistent protein production. Agrobacterium or biolistics-mediated delivery of transgenes.	Potential for large-scale production. Suitable for biologics with high demand or requiring complex PTMs. The use of edible plant species for oral delivery and cereals for long storage at ambient temperature.	The time-consuming process of plant transformation and regeneration. Relatively lower protein yield. Potential position effect and gene silencing. Regulatory considerations and public concerns regarding GMOs.
Stable chloroplast transformation [153,154,155]	Each plant cell has 10,000 copies of the chloroplast genomes, which can stably integrate the gene of interest using a biolistic method of delivery.	The recombinant protein can be expressed at very high levels, up to 45% of the TP; there is no reported gene silencing; toxic proteins can be expressed successfully; more than one gene can be expressed, facilitating the production of complex proteins; and no gene flow.	A time-consuming process with low transformation frequencies, the formation of inclusion bodies, and challenges during the purification of recombinant proteins. Regulatory considerations.
Viral Vectors [156]	Utilization of viral vectors, such as TMV or CPMV, to enhance protein expression levels by leveraging the viral replication machinery within plants.	Increased protein yields compared to non-viral expression systems. Compatibility with both transient and stable expression approaches.	Risk of viral contamination and potential biosafety concerns. Additional steps are required for viral vector construction and handling. Potential for adverse effects on plant growth.
Transient Expression [157,158,159]	Rapid production of target proteins by introducing the gene of interest into plants using binary vector-based plasmids and agroinfiltration or viral vectors.	Quick and high-yield protein production. Suitable for rapid response scenarios. Flexibility and versatility in terms of the biological molecules that can be produced.	The transient nature of expression requires repeated plant agroinfiltration for continuous production.
Plant cell cultures [160]	Production of recombinant proteins in plant cell suspension cultures.	Potential for easy scale-up for manufacturing under aseptic conditions using classical fermentation technology. Low risk of contamination. The same regulatory requirements as mammalian cell production systems.	Slower growth and lower yields compared to microbes and mammalian cells; overall cost is medium. Plant cell cultures are characterized by heterogenicity.
Hairy roots *Rhizobium rhizogenes* [161]	Rhizosecretion of recombinant proteins in the hydroponic medium.	Secretion of the proteins into medium, facilitated purification, and improved product homogeneity.	Protein degradation, high proteolytic activity, and GMO regulatory considerations.
Gene Editing [162]	Precise modification of plant genomes using gene editing technologies, such as CRISPR/Cas9, to optimize protein production and reduce proteolytic degradation.	Targeted modification of specific genes or regulatory elements to enhance protein expression. Potential for multiplex gene editing to improve multiple traits simultaneously.	Technical complexity and optimization required for gene editing experiments. Potential for off-target effects and unintended genomic modifications. Regulatory considerations for GMOs.
Glycoengineering [163]	Elimination of unwanted glycan modifications and expression of glycosylation enzymes to provide the required specific glycans.	Production of recombinant glycoproteins with human-type glycans that resemble natural glycosylation. Eliminate unwanted glycan modifications.	Some plant species do not tolerate the engineering of glycan processing pathway, N-glycan heterogenicity, or GMO safety risks.
Downstream Processing [164,165]	Implementation of purification strategies to effectively remove plant-specific contaminants, ensuring stability and quality of the final product.	Improved purity and removal of unwanted plant-specific contaminants. Optimization of downstream processing for specific biological molecules.	Additional processing steps, costs, and requirements. Need for customized purification methods for different biological molecules.
Formulation and Delivery [166,167]	Development of innovative formulation and delivery methods to improve stability, bioavailability, and targeted delivery of plant-derived biologics.	Improved stability during storage and transportation. Enhanced bioavailability and efficacy in the target tissues or cells. Targeted delivery to specific organs or cellular compartments.	Additional costs associated with formulation and delivery systems. Potential challenges in achieving targeted delivery to specific sites.

**Table 4:** An overview of regulatory considerations for plant-derived biologics. It includes information on the plant expression system, genetic modifications, and the characterization of biologics. The variability in expression and management of co-expression.

Regulatory Considerations for Plant-Derived Biologics
1. Source of plant expression system: Detailed information on plant species, genetic modifications, and expression vectors used in production.
2. Characterization of biologics: Thorough evaluation of structure, purity, potency, and stability.
3. Variability in expression: Measures to ensure consistent production and quality of biologics despite inherent variability within plants.
4. Co-expression of plant-specific proteins/allergens: Identification and mitigation of potential risks associated with unintended co-expression.
5. Environmental impact: Risk assessment to evaluate the potential environmental effects of cultivation and production processes.
6. Case-by-case evaluation: Tailoring regulatory requirements based on the specific characteristics of each plant-derived biologic.
7. Global harmonization: Collaboration between regulatory authorities and industry stakeholders to establish international guidelines and standards.
8. Good Manufacturing Practice: Compliance with GMP guidelines to ensure consistent quality and safety during manufacturing processes.
9. Preclinical and clinical data: Submission of comprehensive preclinical and clinical data to establish safety and efficacy profiles of plant-derived biologics.
10. Post-marketing surveillance: Monitoring and reporting of adverse events and safety data following the commercialization of plant-derived biologics.
11. Intellectual property rights: Consideration of intellectual property protection for novel plant-derived biologics and their manufacturing processes.
12. Labeling and product information: Clear and accurate labeling to provide information on indications, dosage, administration, and potential risks associated with the use of plant-derived biologics.
13. Risk management plan: Development of a risk management plan to identify and address potential risks throughout the lifecycle of plant-derived biologics.
14. Regulatory updates and advancements: Staying informed about evolving regulations, guidelines, and advancements in the field of plant-derived biologics.

## Data Availability

Not applicable.
